# Psychosomatic symptoms and stressful working conditions among Palestinian nurses: a cross-sectional study

**DOI:** 10.1080/10376178.2016.1188018

**Published:** 2016-05-27

**Authors:** Yousef Jaradat, Khaldoun Nijem, Lars Lien, Hein Stigum, Espen Bjertness, Rita Bast-Pettersen

**Affiliations:** ^a^ Department of Occupational Medicine and Epidemiology, National Institute of Occupational Health, Oslo, Norway; ^b^ Section for Preventive Medicine and Epidemiology, Institute of Health and Society, University of Oslo, Oslo, Norway; ^c^ Occupational Epidemiology and Biological Research Lab, Department of Biology, Hebron University, Hebron, Occupied Palestinian Territory; ^d^ National Competence Centre for Dual Diagnosis, Innlandet Hospital Trust, Hamar, Norway

**Keywords:** nurses, psychosomatic symptoms, stressful working conditions, gender differences

## Abstract

*Background:* High levels of perceived stressful working conditions have been found to have an adverse effect on physical and mental health. *Objectives:* To examine the associations between self-reported stressful working conditions and Psychosomatic Symptoms (PSS), and to investigate possible gender differences. *Methods:* The present cross-sectional study comprises 430 nurses employed in Hebron district, Palestine. Self-reported stressful working conditions were recorded, and a Psychosomatic Symptoms Check list was used to assess prevalence of PSS. *Findings:* Median score on the psychosomatic symptom checklist for the group was 11, (range 1–21). Women reported more symptoms than men, with medians 11.6 and 10.0, respectively (*p* = .0001). PSS were associated with more self-reported stressful working conditions for both men (*p* < .0001) and women (*p* < .0001). The association was strongest among men. *Conclusions:* PSS were associated with high self-reported stressful working conditions, and this association was strongest among the men.

High levels of perceived stressful working conditions have been found to have an adverse effect on the physical and mental health of nurses (Mc Vicar, 2003; Mojoyinola, 2008). Nurses in clinical work may experience adversity and moral distress that inhibit their capacity to provide morally sensitive patient care (Vanderheide, Moss, & Lee, 2013). Occupational stressors among nurses include a lack of perceived social support from supervisors and peers (Bartram, Joiner, & Stanton, 2004; Sveinsdóttir, Biering, & Ramel, 2006), low levels of work conditions and caring for dying patients (Hamaideh, Mrayyan, Mudallal, Faouri, & Khasawneh, 2008), working overtime and non-organizational factors (Mc Vicar, 2003) and shift work, with round the clock care for patients (Hamaideh & Ammouri, 2011; Jaradat et al., 2012).

In the literature, the terms “stressors”, “stressful events” and “stress” are used in several ways (Folkman & Lazarus, 1991). Potter and Fiedler (1981) define the term “stress” as the result of two divergent forces that act on the individual. As the demands begin to outstrip the individual’s resourses, the individual will experience a corresponding increase in stress. In the present study, we will use the terms “stressor”, “stressful event” and “self-reported or perceived stressful working conditions or work situation” to mean an environmental condition or a stimulus to which the individual is exposed. “Stress” will be used as a perceived bodily response to a stressful event/stressor (Beck & Srivastava, 1991; Cohen & Wills, 1985; Martins, Moraes Ferreira, & Guilhem, 2013).

Nurses are exposed to stressors with potential adverse effects on physical and psychological health (Dickinson & Wright, 2008; Jaradat et al., 2012). High levels of work stress among nurses may result in increasing job-related accidents, late arrivals and absence of work, and may thus result in decreased productivity and responsibility (Lee & Wang, 2002) and may affect the nurses’ professional efficiency, which might reduce the quality of patient care (Kane, 2009; Kawano, 2008; Lindegård, Larsman, Hadzibarjramovic, & Ahlborg, 2014; Sherman, 2004). Health care staff are being faced with increasing pressure, and emotional exhaustion scores were high among Hungarian health care staff included nurses (Pikó, 2006). Several studies have reported an association between stressful working conditions on the one hand and psychosomatic symptoms (PSS) and/or musculoskeletal complaints on the other hand.

The incidence of psychosomatic disorders was increased in Indian hospital nurses who reported higher self-reported stress scores; stomachache, back pain, and stiffness of shoulders and neck were related to exposure to stressors at home and the workplace (Kane, 2009). A study on the prevalence of musculoskeletal complaints among nurses reported that nurses had back complaints (36%), arm and neck complaints (30%), and leg complaints (16%); in addition, most of the nurses (89%) considered nursing work as physically strenuous (Engels, Van der Gulden, Senden, & van't Hof, 1996). A 44% prevalence of back disorders among female nurses was reported (Violante et al., 2004).

Fewer years of experience and negative family support are predictors of psychological symptoms among nurses (Arafa, Nazel, Ibrahim, & Attia, 2003). Several studies have reported that women face different stressors than men (Artazcoz et al., 2004; Gyllensten & Palmer, 2005) and that they may react in different ways to perceived stressors. A study among workers in Spain found that men’s health status and PSS were not associated with family demands but were associated with occupational social class; however, in women, PSS were associated with family and job demands (Artazcoz et al., 2004). The most frequent PSS among both women and men were sleeping problems, chronic fatigue and back pain, whereas tension headaches and chronic fatigue were the most frequent PSS among women (Pikó, Barabás, & Boda, 1997).

In a study conducted in one hospital in Palestine, nurses reported that they faced harsh treatment from the public because they are in direct daily contact with patients and their families; in addition, they reported that supervision focuses on mistakes rather than on providing a real evaluation of the quality of their work and that punishment is the most common way in which their errors are addressed (Hasan-Bitar & Narrainen, 2011). Palestinians are at a high risk of exposure to traumatic events, which have the capacity to produce traumatic stress reactions (Khamis, 2005). A previous study reported a major prevalence of depressive episodes of 18.7% and a prevalence of post-traumatic stress disorders of 26.5% among adult Palestinians (Madianos, Sarhan, & Koukia, 2011).

Health care and services in Palestine are provided by the Ministry of Health (MOH), Non**-**Governmental Organizations, United Nations Relief and Works Agency (UNRWA), and private medical service. Most of health care services in Hebron city are primary health care centers and hospitals. The primary health care centers provide the general health examination, community care, maternity-health child care, and school health care. The hospitals include an assortment of operations and procedures in medicine. There are 51 hospitals in West Bank (WB) and 25 in Gaza Strip (GS) with total number of 5108 beds, this comprises both government and non-government hospitals, and this means that there are 12.6 beds per 10,000 of population. After the second intifada (2000) and its political consequences including poverty (53%) and unemployment (30%) (Palestinian Central Bureau of Statistics [PCBS], 2008), Palestinian patients tend to consult the governmental hospitals and clinics through the free health insurance offered by the MOH. A total of 11,300 nurses (6618 in WB and 4682 in GS) are supervised and regulated by the Palestinian Nursing Association. It is estimated that there is one nurse for one thousand inhabitants (1/1000) in Hebron district which reflects the shortage in nurses’ labor force and the huge work load. In the primary health care centers, nurses work morning shift, while in hospitals the nurses are working day, evening, and night shifts. To our knowledge, this study is the first to report on PSS among nurses in Palestine. This study is part of a larger study on the health of Palestinian nurses.

The aim of this study was to examine the associations between self-reported stressful working conditions and PSS among nurses in Palestine. Because there are a much larger proportion of male nurses in Palestine than in Western countries, we also aimed to investigate possible gender differences in the association between perceived stressful working conditions and PSS.

## Methods

### Study population

The present cross-sectional study involved nurses working in the Hebron district of Palestine. A total of 542 nurses were working at hospitals and primary health care centers in the autumn of 2008. They were employed in various health care settings including the governmental sector and others (non-governmental, UNRWA and private sectors). The data were collected from August to October, 2008. The inclusion criterion was at least one year of service prior to the start of the study. Of the 542 employed nurses, 70 did not meet this criterion; 18 nurses were not available because they were on leave, resulting in 454 available nurses. Ten refused to participate, and two nurses with incomplete background data were excluded. We also excluded 12 nurses who did not answer the questionnaires on self-reported stressful working conditions and PSS. After all the exclusions, 430 nurses were included in the analysis, resulting in a participation rate of 94.7%. As shown in Table 1, the dataset comprised 259 (60%) women and 171 (40%) men, ranging in age from 20 to 59 years (mean age 33.6, SD 8.6 years). Most participants (74.4%) were married, (42.3%) had 15−16 years of education, and (71.8%) had 7 or more years of experience.

**Table 1.** Self-reported perceived level of stressful working conditions/work situations among nurses by socio-demographics and organizational characteristics (*N* = 430).

	Low		Medium		High	
Variables	*N*	%	*N*	%	*N*	%
	53	12.3	204	47.4	173	40.2
Females	26	10.0	127	49.0	106	41.0
Males	27	15.8	77	45.0	67	39.2
*Age group (years)*						
<30	17	10.1	80	47.6	71	42.3
30–45	28	13.4	99	47.4	82	39.2
>45	8	15.1	25	47.2	20	37.7
*Marital status*						
Married	42	13.1	155	48.4	123	38.4
Un married	11	10.0	49	44.6	50	45.4
*Number of children*						
No child	18	12.0	69	46.0	63	42.0
1–3 child	14	10.8	62	47.7	54	41.5
>3 child	20	13.7	73	50.0	53	36.3
*Family size*						
1–4	9	07.4	60	49.6	52	43.0
5–8	31	14.7	98	46.5	82	38.9
>8	12	13.6	42	47.7	34	38.6
*Years of education*						
10–14	18	09.1	88	44.7	91	46.2
15–16	27	14.8	97	53.3	58	31.9
>16	8	15.7	19	37.3	24	47.1
*Providing financial support to extended family members*						
No	19	13.4	78	54.9	45	31.7
Yes	34	11.8	126	43.8	128	44.4
*Work setting*						
Government	37	16.0	105	45.3	90	38.8
Others	16	08.1	99	50.0	83	41.9
*Work schedule*						
Morning only	31	16.3	97	51.1	62	32.6
Evening or night only	3	14.3	10	47.6	8	38.1
Alternating	19	08.7	96	44.0	103	47.3
*Type of task*						
Direct patient care	39	11.1	164	46.7	148	42.2
Indirect patient care	7	20.6	14	41.2	13	38.2
Both types of care	7	15.6	26	57.8	12	26.7
*Experience (years)*						
1–6	13	11.0	60	50.9	45	38.1
7–12	23	11.6	90	45.5	85	42.9
>12	14	13.7	50	49.0	38	37.3
*Work hours (week)*						
<36	23	13.0	88	49.7	66	37.3
36–44	23	20.4	52	46.0	38	33.6
>44	7	05.0	64	45.7	69	49.3
*Work overtime*						
No	42	12.4	169	49.9	128	37.7
Yes	11	12.2	35	38.9	44	48.9

### Data collection

Background data were collected using a predesigned self-administered questionnaire. The questionnaire included questions about socio-demographic and organizational characteristics variables. To measure the exposure variable, the self-reported perceived stressful working condition/situation was assessed by using the question, “How stressful do you perceive your present job?” (Pikó, 1999; Potter & Fiedler, 1981). The responses were ranked on a nine-point scale from the lowest stress level (1) to the highest stress level (9), due to small numbers in some of the nine levels, we performed the analyses based on three exposure categories; low stressful working conditions level (a score of 1−3), a medium stressful working conditions level (scores of 4−6) and a high stressful working conditions level (scores of 7−9). To measure the outcome variable, a psychosomatic symptom questionnaire was used to record the prevalence of self-reported PSS. The questionnaire comprised a scale that has been applied in several studies (Pikó, 1999, 2006; Pikó et al., 1997), and with a reported Cronbach’s *α* of 0.80 (Pikó, 2006). Seven symptom items were queried: back pain, tension headache, sleeping problems, chronic fatigue, stomach acidity, tension diarrhea, and heart palpitation. This measure was used in order to obtain information on the frequency of these symptoms during the last 12 months. For example, respondents were asked: “During the last 12 months, how often did you have back pain?” Responses were coded as follows: never (0), seldom (1), occasionally (2), and often (3). A Likert-type scoring procedure of 0, 1, 2, and 3 was applied to investigate the association of PSS with self-reported stressful working conditions, allowing for a total score range between 0 and 21 and was reliable with a Cronbach’s *α* of 0.78; where a higher score represents increased symptom occurrences.

The questionnaires were translated from English into Arabic by the research team and were revised with assistance from a professional translator in the field to overcome language problems. The questionnaires were piloted prior to the formal data collection by randomly selecting 24 nurses. After piloting, the questions were amended and rephrased in order to be clearer. Back translation was not performed.

### Ethical considerations

The study was approved by the Regional Committee for Medical and Health Research Ethics, REC South East, Norway. Permission to conduct the study was obtained from the Palestinian Ministry of Health and other health care providers (non-governmental, UNRWA and private sectors), and the research protocol was approved by the research board at Hebron University. The participants were provided with information about the purposes of the study and were informed that the collected data were strictly confidential and would be used for scientific purposes only. Additionally, they were informed that their participation was voluntary, that they could refuse to answer any item, and that there would be no adverse consequences for refusing to participate. Informed written consent was obtained from each participant prior to beginning the study.

### Statistical analysis

The statistical analysis package STATA (Version 10.0) was used for the analyses. The exposure variable was self-reported perceived stressful working conditions. PSS were used as the outcome variable. In the first part, the PSS were used as a continuous variable on the 0−21 scale. We inspected the distribution in density plots, tabulated the median levels with non-parametric tests (Kruskal–Wallis) in the bivariate analysis, and used an ordinary linear regression in the multivariable analysis. The regression model was built with a three-level stressful working condition scale as the main independent variable, and the overall scores of PSS were used as the dependent variables. The following items regarding the socio-demographics and organizational characteristics were considered as possible confounders and were included in the primary analysis: gender, age, marital status, number of children, family size, education, providing financial support to extended family members (other than children and spouse), work setting, work schedule, type of task, years of experience, work hours per week, and work overtime. Factors that changed the association less than 10% between stressful working conditions level and PSS were removed. The variables age, years of education, working overtime, and providing financial support to extended family members were included as confounding variables in the final model. Because PSS vary with gender, most analyses were performed separately for females and males. To examine whether the effects of self-reported levels of stressful working conditions were different for men than for women, we included an interaction term.

The psychosomatic symptom scale is constructed from seven underlying items on a four-unit scale. In the second part of the analysis, we focused on these individual items as outcomes. We showed the prevalence of the psychosomatic items based on levels of stressful working conditions in the bivariate analysis. We then dichotomized each item using the levels “often” as 1 and “occasionally”, “seldom” or “never” as 0. Linear binomial regression models were used to estimate the associations between the occurrence of PSS (level “often”) and the self-reported level of stressful working conditions. These models show prevalence differences (PDs) as association measures. Age, education, worked overtime, and providing financial support to extended family members were included as confounders. Separate models were used for each gender (14 models in all). In some cases, the models did not converge, so we used a linear regression model with robust variance estimation as an alternative. The level of statistical significance was defined as *p* ≤ .05 with a 95% confidence interval (CI).

The assumptions of the linear model (linearity and constant error variance) were checked by plotting residuals against the predicted values (i.e. by adding a smoothing curve with a CI). We checked the robustness of the model by plotting delta-beta values for the exposure variables. The regression model showed no deviation from the assumptions of linearity and the constant variance of the residuals. The results are therefore considered robust against outliers.

## Results

**Figure 1.**
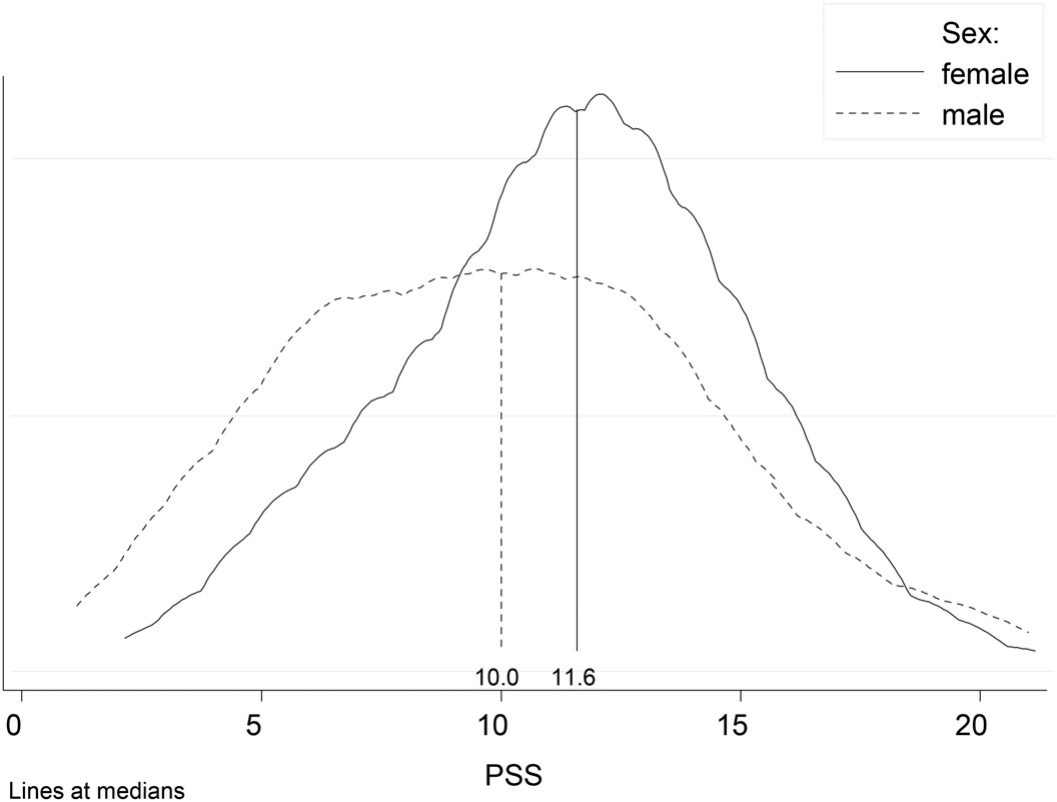
Distribution of PSS among nurses by gender (vertical lines indicate medians).

The median score of PSS for the nurses as a group was 11.0, ranging from 1 to 21. Figure 1 shows the distribution of PSS for the female and male nurses, with lines indicating the medians. The women reported more symptoms than the men: 11.6 versus 10.0 (*p* = .0001). The entire distribution was shifted to the right compared with the males. However, the variance among the males was higher, so there were slightly more males with very high symptom scores (>18).

Table 1 shows the self-reported level of stressful working conditions among nurses by socio-demographics and working characteristics. 12.3% of the nurses reported low stressful working conditions, 47.4% reported medium stressful working conditions, and 40.2% reported high stressful working conditions. A significant association was found between high self-reported stressful working conditions and education, providing financial support to extended family members, working in settings other than governmental organizations, working alternating shifts, and long working hours per week.

**Figure 2.**
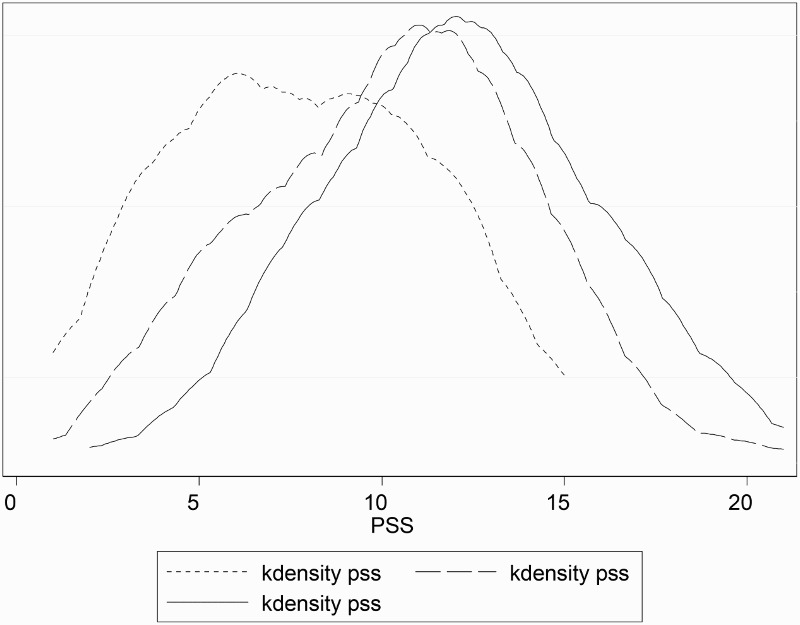
Distribution of PSS among nurses by self-reported level of perceived stressful working conditions. Legend: short dash line (PSS), long dash line (PSS), and solid line (PSS) indicate low, medium and high levels of perceived stressful working conditions, respectively.

**Figure 3.**
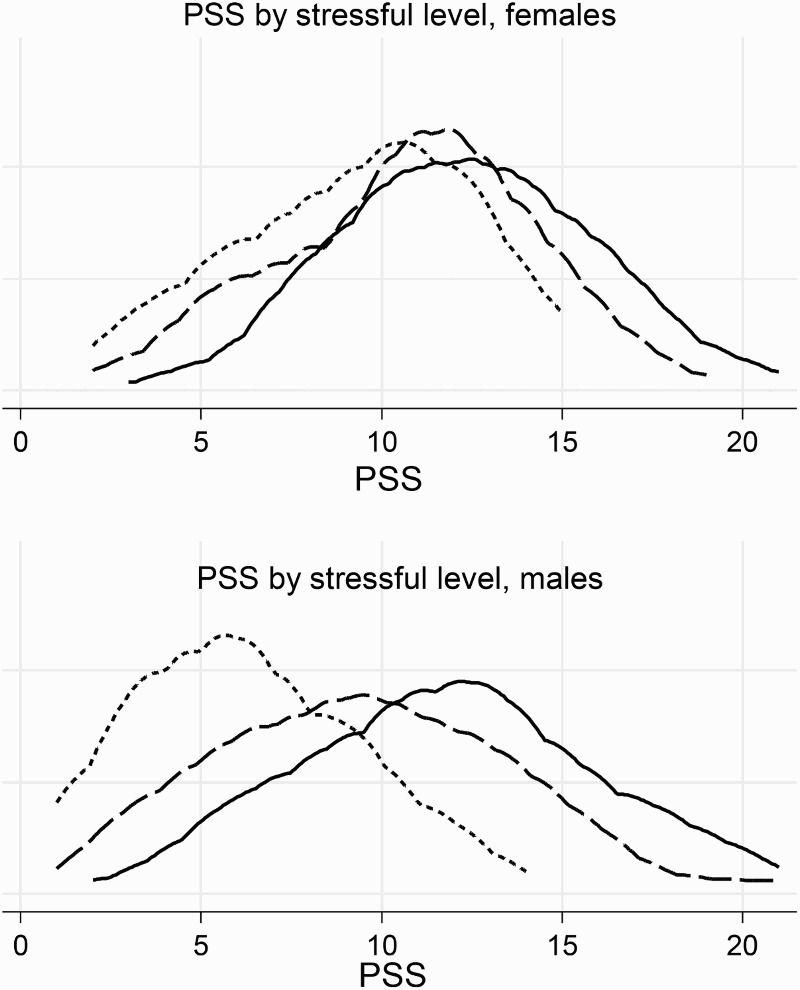
Distribution of PSS among nurses by self-reported level of stressful working conditions and gender. The upper panel shows females, and the lower panel shows males: short dash line (PSS), long dash line (PSS), and solid line (PSS) indicate low, medium and high levels of perceived stressful working conditions, respectively.

Figures 2 and 3 illustrate the distribution of PSS related to the self-reported level of stressful working conditions for all nurses and separately for each gender, respectively. PSS were more prevalent among nurses with high self-reported stressful working conditions. The distribution of PSS for women and men suggests a stronger association with perceived stressful working conditions for men than for women (Figure 3). This pattern is shown in Table 2. The median scores were 12 units among both women and men in association with high self-reported stressful working conditions. In contrast, the median symptom scores in association with low self-reported stressful working conditions were 10 units among women and only 5.6 units among men. There were also gender differences with regard to the influence of socio-demographics or organizational characteristics on PSS. For women, the median symptom score was higher for those with smaller families, and for those who worked overtime. For men, the median score of symptoms was higher for married men, for men with 1–3 children and men who had more years of education. Men who worked with both direct and indirect patient care, and had seven or more years of work experience also reported more symptoms (Table 2).

**Table 2.** Medians of PSS by self-reported level of stressful working conditions, socio-demographics and work characteristics among nurses by gender.

	Female				Male			
	*N*	%		*p-*Value	*N*	%		*p-*Value
Variables	259	60	Median score		171	40	Median score	
Socio-demographic factors								
*Self-reported level of stressful working conditions*								
Low	26	10.1	10.0	<0.001	27	15.9	05.6	<0.001
Medium	127	49.0	11.0		77	44.7	09.0	
High	106	40.9	12.0		67	39.4	12.0	
*Age group (years)*								
<30	92	35.5	11.2	0.62	76	44.7	08.5	0.25
30–45	135	52.1	11.8		74	43.5	11.0	
>45	32	12.4	11.5		20	11.8	10.0	
*Marital status*								
Married	195	75.3	12.0	0.43	124	72.9	10.0	0.03
Un married	64	24.7	11.0		46	27.1	08.0	
*Number of children*								
No child	88	34.4	12.0	0.65	62	36.7	08.0	0.04
1–3 child	79	30.9	11.0		51	30.2	11.0	
>3 child	89	34.8	11.4		56	33.1	10.0	
*Family size*								
1–4	87	34.1	12.0	0.02	34	20.7	10.5	0.26
5–8	110	43.1	11.0		100	61.1	11.0	
>8	58	22.7	11.0		30	18.3	08.0	
*Years of education*								
10–14	129	49.8	11.8	0.16	67	39.4	09.0	0.04
15–16	104	40.2	11.0		78	45.9	10.0	
>16	26	10.0	13.0		25	14.7	12.0	
*Providing financial support to extended family members*								
No	103	39.8	11.0	0.15	38	22.4	11.0	0.08
Yes	156	60.2	12.0		132	77.6	09.0	
Organizational characteristics								
*Work setting*								
Government	141	54.4	11.8	0.57	90	52.9	10.0	0.76
Others	118	45.6	11.0		80	47.1	10.0	
*Work schedule*								
Morning only	141	54.7	11.0	0.34	48	28.2	09.0	0.63
Evening or night only	5	01.9	11.0		16	09.4	10.0	
Alternating	112	43.4	12.0		106	62.4	10.0	
*Type of task*								
Direct patient care	211	81.5	11.0	0.37	139	81.8	10.0	0.05
Indirect patient care	15	05.8	11.0		19	11.2	07.0	
Both types of care	33	12.7	12.0		12	07.1	12.0	
*Experience (years)*								
1–6	65	25.9	11.0	0.47	53	31.9	08.0	0.01
7–12	119	47.4	12.0		79	47.6	11.0	
>12	67	26.7	11.0		34	20.5	10.0	
*Work hours (week)*								
<36	114	44.0	12.0	0.99	63	37.1	10.0	0.43
36–44	73	28.2	11.0		39	22.9	08.5	
>44	72	27.8	11.0		68	40.0	11.0	
*Work overtime*								
No	222	85.7	11.0	0.02	116	68.6	09.5	0.20
Yes	37	14.3	12.0		53	31.4	10.0	

*p*-Values for difference of medians are based on Kruskal–Wallis test (*N* = 430).

The gender differences adjusted for age, education, working overtime, and providing financial support to extended family members are illustrated in Table 3, showing a significant association between perceived stressful working conditions and symptoms, and this association was much stronger for men than for women. Men with high self-reported stressful working conditions scored 5.40 units higher than men with low self-reported stressful working conditions (95% CI 3.60−7. 20), while women with high self-reported stressful working conditions scored 3.00 units higher than women with low self-reported stressful working conditions (95% CI 1.50–4. 50). Furthermore, while working overtime was associated with more symptoms for women, the male nurses with more education reported more symptoms. On the other hand, the men who financially supported extended family members showed a 1.70 unit lower score (95% CI −3.10 to −0.26). To examine whether the effects of stressful levels of working conditions were different for men than for women, we included an interaction term. The psychosomatic symptom score in association with high self-reported stressful level rose with 3.2 units (95% CI 1.6–4.8) for women and with 5.5 units (95% CI 3.8–7.1) for men (*p* = .05) (not tabulated).

**Table 3.** Association between overall PSS and socio-demographics or organizational characteristics.

	Female		Male	
Variables	*Adjusted coefficient	95% (CI)	*Adjusted coefficient	95% (CI)
*Self-reported level of stressful working conditions*				
Low stressful level	Reference		Reference	
Medium stressful level	1.59	0.10–3.08	3.29	1.57–5.00
High stressful level	3.00	1.50–4.50	5.40	3.60–7.20
*Age group (years)*				
<30	Reference		Reference	
30–45	0.10	−0.88–1.10	0.88	−0.38–2.14
˃45	−0.45	−1.91–1.00	1.21	−0.80–3.23
*Years of education*				
10–14	Reference		Reference	
15–16	0.38	−0.54–1.30	−0.13	−1.46–1.20
>16	1.40	−0.07–2.90	2.00	0.16–3.80
*Providing financial support to extended family members*				
No	Reference		Reference	
Yes	0.38	−0.53–1.30	−1.70	−3.10 to −0.26
*Work overtime*				
No	Reference		Reference	
Yes	1.35	0.06–2.63	1.20	−0.12–2.50

Note: CI, confidence interval.

*Adjusted for age, education, working overtime and providing financial support to extended family members.

Table 4 shows the frequency of the different psychosomatic items across categories of self-reported stressful working conditions. There was a gender difference with respect to the type of reported symptoms. Among the women who reported high stressful working conditions, 45.3% reported back pain, while this symptom was reported by 31.3% of the men. Tension headache was reported by 36.8% of the women with high perceived stressful working conditions, compared with 26.9% of the men with high perceived stressful working conditions. On the other hand, among the nurses with high perceived stressful working conditions, 37.9% of the men and 19.8% of the women reported sleeping problems, and 23.9% of the men and 17.5% of the women reported stomach acidity.

**Table 4.** Prevalence/frequency of the different PSS among nurses by gender and by level of self-reported stressful working conditions (*N* = 430).

	Females			Males		
	Low	Medium	High	Low	Medium	High
*Back pain*						
Often	38.5	38.6	45.3	11.1	17.1	31.3
Occasionally	42.3	52.0	48.1	33.3	55.3	52.2
Seldom	07.7	07.9	05.7	33.3	18.4	14.9
Never	11.5	01.6	00.9	22.2	09.2	01.5
*Tension headache*						
Often	11.5	18.1	36.8	00.0	09.2	26.9
Occasionally	61.5	61.4	51.9	44.4	55.3	59.7
Seldom	26.9	17.3	11.3	37.0	25.0	10.4
Never	00.0	03.1	00.0	18.5	10.5	03.0
*Chronic fatigue*						
Often	03.8	19.4	29.2	00.0	06.7	20.9
Occasionally	46.2	42.7	42.5	11.1	44.0	49.3
Seldom	26.9	25.8	23.6	40.7	25.3	20.9
Never	23.1	12.1	04.7	48.1	24.0	09.0
*Sleeping problems*						
Often	07.7	18.9	19.8	03.8	09.5	37.9
Occasionally	38.5	40.9	55.7	11.5	41.9	31.8
Seldom	46.2	29.1	20.8	53.8	31.1	27.3
Never	07.7	11.0	03.8	30.8	17.6	03.0
*Stomach acidity*						
Often	00.0	12.7	17.5	07.4	17.3	23.9
Occasionally	30.8	36.5	35.0	29.6	44.0	29.9
Seldom	42.3	26.2	29.1	25.9	22.7	31.3
Never	26.9	24.6	18.4	37.0	16.0	14.9
*Tension diarrhea*						
Often	00.0	02.4	07.5	00.0	04.0	04.6
Occasionally	19.2	12.7	17.9	03.7	13.3	15.4
Seldom	30.8	29.4	34.9	29.6	22.7	35.4
Never	50.0	55.6	39.6	66.7	60.0	44.6
*Palpitation*						
Often	00.0	06.3	10.4	00.0	02.7	09.2
Occasionally	26.9	31.0	38.7	18.5	32.4	35.4
Seldom	26.9	35.7	34.0	25.9	35.1	27.7
Never	46.2	27.0	17.0	55.6	29.7	27.7

Table 5 shows the association between a high score on the separate psychosomatic items and self-reported stressful working conditions in a multivariable analysis. The first model shows the association between “often back pain” and self-reported stressful working conditions. Women with high self-reported stressful working conditions had a 6-percentage-point (pp) higher prevalence of “often back pain” than women with low stressful working conditions, although this finding was not significant. Men with high stressful working conditions had a 19-pp higher prevalence of frequent back pain than men with low stressful working conditions, a difference that was statistically significant (95% confidence level from 3% to 36%). Among both women and men, a significant association was found between high self-reported stressful working conditions and frequent occurrence of tension headache, chronic fatigue, stomach acidity, tension diarrhea, and palpitation. Among men, associations were also found between high self-reported stressful working conditions and sleeping problems.

**Table 5.** Linear binomial regression model estimating the association between level of self-reported stressful working conditions (low, medium, high) and frequency (often) of the different/individual PSS among female and male nurses.

	Female		Male	
	Crude PD^a^ (95% CI)	Adjusted PD^b^ (95% CI)	Crude PD^a^ (95% CI)	Adjusted PD^b^ (95% CI)
*Often back pain*				
Low stressful	Reference	Reference	Reference	Reference
Medium stressful	0.00 (−0.20–0.20)	0.00 (−0.21–0.20)	0.06 (−0.09–0.21)	0.05 (−0.10–0.20)
High stressful	0.07 (−0.14–0.28)	0.06 (−0.20–0.21)	0.20 (0.04–0.36)	0.19 (0.03–0.36)*
*Often tension headache*				
Low stressful	Reference	Reference	Reference	Reference
Medium stressful	0.07 (−0.08–0.21)	0.06 (−0.08–0.20)	0.09 (0.03–0.16)	0.08 (0.01–0.15)*
High stressful	0.25 (0.10–0.41)	0.23 (0.08–0.39)*	0.27 (0.16–0.38)	0.25 (0.14–0.36)*
*Often chronic fatigue*				
Low stressful	Reference	Reference	Reference	Reference
Medium stressful	0.15 (0.05–0.25)	0.15 (0.04–0.26)*	0.07 (0.01–0.12)	0.05 (−0.01–0.12)
High stressful	0.25 (0.14–0.37)	0.23 (0.10–0.35)*	0.21 (0.11–0.31)	0.20 (0.11–0.30)*
*Often sleeping problems*				
Low stressful	Reference	Reference		Reference
Medium stressful	0.11 (−0.01–0.24)	0.09 (−0.04–0.22)	0.06 (−0.04–0.15)	0.05 (−0.05–0.15)
High stressful	0.12 (−0.01–0.25)	0.07 (−0.07–0.21)	0.34 (0.20–0.47)	0.32 (0.19–0.46)*
*Often stomach acidity*				
Low stressful	Reference	Reference	Reference	Reference
Medium stressful	0.13 (0.07–0.18)	0.14 (0.07–0.21)*	0.10 (−0.03–0.23)	0.09 (−0.04–0.22)
High stressful	0.17 (0.10–0.24)	0.15 (0.06–0.24)*	0.16 (0.02–0.31)	0.16 (0.01–0.31)*
*Often tension diarrhea*				
Low stressful	Reference	Reference	Reference	Reference
Medium stressful	0.02 (−0.00–0.05)	0.03 (−0.00–0.05)	0.04 (−0.01–0.08)	0.04 (−0.01–0.09)
High stressful	0.08 (0.03–0.13)	0.08 (0.02–0.14)*	0.04 (−0.01–0.10)	0.05 (0.00–0.10)*
*Often palpitation*				
Low stressful	Reference	Reference	Reference	Reference
Medium stressful	0.06 (0.02–0.11)	0.07 (0.02–0.12)*	0.03 (−0.01–0.06)	0.02 (−0.02–0.06)
High stressful	0.10 (0.05–0.16)	0.12 (0.05–0.18)*	0.09 (0.02–0.16)	0.09 (0.02–0.16)*

Note: CI, confidence interval.

^a^Crude PD for age, education, working overtime and providing financial support to extended family members.

^b^Adjusted PD for age, education, working overtime and providing financial support to extended family members.

*Statistical significant.

## Discussion

The main finding in this study was an association between self-reported stressful working conditions and an increased number of PSS. To our knowledge this is the first available study of PSS among nurses where 40% of the nurses were males, allowing to study gender differences with respect to such symptoms. The results indicate gender differences; the female nurses reported more symptoms, but the association between self-reported stressful working conditions and symptoms was stronger for the men (interaction term *p*-value = .05).

The finding that PSS were more prevalent among nurses with high self-reported stressful working conditions than among nurses with low self-reported stressful working conditions is consistent with previous studies that reported that a high level of exposure to stressors contributes to some forms of PSS (Kane, 2009; Kawano, 2008; Mojoyinola, 2008; Pikó, 1999). Higher levels of stressful working conditions among nurses were associated with more health complaints such as back pain, chronic fatigue, tension headache, and sleeping problems (Pikó, 1999). Most nurses are involved in direct patient care that includes lifting and transporting patients and standing for long hours, and this may produce psychosomatic disorders. A study by Kawano (2008) stated that hospital nurses with excessive amounts of work under pressure and difficulties with caring for patients with various complaints may be more likely to feel physical and mental exhaustion, and this might affect patient satisfaction. Several studies have shown a connection between stressful working conditions and the physical and mental health of nurses (Hamaideh et al., 2008; Mojoyinola, 2008; Pikó, 1999).

### Gender differences

The finding of more PSS reported by women than men is consistent with other studies. From a study with the same psychosomatic questionnaire as the one used in the present study, the mean score of PSS was higher among female compared to male students (Pikó et al., 1997). Female patients displayed more head-related PSS than males (Tsai, 2010). The higher frequency of PSS among women might be explained because women have extra responsibilities with housekeeping duties and caring for children in addition to the tasks in the workplace. House work adds to the physical work load and increases the incidence of musculoskeletal disorders among working women (Kishi, Kitahara, Masuchi, & Kasai, 2002; Scott, Hwang, & Rogers, 2006). An interaction between work and family roles resulting in chronic fatigue among female nurses may serve as a risk factor especially when combined with the acute fatigue associated with night work (Clissold, Smith, Accutt, & Di Milia, 2002). The conflict between career and family life roles may have a stronger negative impact on the physical and mental well-being of women (Artazcoz et al., 2004; Bourbonnais, Comeau, & Vézina, 1999).

The finding that male workers seemed to be more vulnerable to self-reported perceived stressful working conditions is an unexpected finding. Other studies do not support this finding (Artazcoz et al., 2004; Gyllensten & Palmer, 2005; Pikó et al., 1997; Pikó, 2006). One explanation could be related to the work situation, a larger percent of the men reported that they had a work schedule with alternating shifts (Table 2). Shift work has been reported to be associated with a variety of health complaints by nurses (Conway, Companini, Sartori, Dotti, & Costa, 2008; Jaradat et al., 2012; Mc Vicar, 2003). Further, in the Palestinian society, male nurses have an obligation to work on-call and in emergency situations, which may account for the effect of stressful working conditions.

Another explanation for the difference might be related to the role expectations in the Palestinian society where men do have a larger economic responsibility for their family. Palestinian data indicates that 87.3% of the women over age 18 do not have to contribute to the family budget, compared to 18% of the men over 18 (PCBS, 1999). Married men with more children reported more symptoms. Palestinian women’s participation in the formal labor force is 16% compared to 66.3% for men (PCBS, 2008). These conditions may increase the expectation for the male nurses to support their family financially, which might aggravate their health conditions and affect the occurrence of psychosomatic disorders.

The finding of fewer PSS among female nurses with a larger family size might indicate that a large family with children represents an emotional support for the women. Palestinians favor marriage and children, and the family forms the center of an individual’s life and affects decisions such as whether or not to marry (Haj-Yahia, 1994).

### The different/individual PSS

In our study, back pain was the most frequently reported symptom in nurses, which is consistent with the findings of other studies (Engels et al., 1996; Kane, 2009; Pikó et al., 1997). Perceived stressful working conditions were found to be strongly associated with low-back pain among Danish female nurses (Gonge, Jensen, & Bonde, 2001). Lifting and transporting patients may lead to back pain among nurses. Headache was the second most frequently reported symptom by nurses in this study. Nurses who worked more than 49 hours a week had a higher incidence of headaches (Callaghan, Tak-Ying, & Wyatt, 2000). A stressful environment is associated with primary headaches in nurses in Taiwan; approximately 50% of the nurses had experienced primary headaches, and 48.1% had episodic – type headache; in addition, a neurological interview revealed that 13.4% had tension headaches (Lin, Huang, & Wu, 2007). Sleeping problems and fatigue were also frequently reported by the nurses. It is possible that shift and night work may have an impact on the sleep and physical health of the nurses; this supports the findings from earlier studies (Bara & Arber, 2009; Lin et al., 2007). Working longer hours may lead to fatigue (Wirtz, Lombardi, Willetts, Folkard, & Christiani, 2012). Having control over work hours can protect against high fatigue (Nijp, Beckers, Geurts, Tucker, & Kompier, 2012).

### Strengths and limitations

#### Strengths

One of the fundamental strengths of the present study is the high response rate (94.7%). We were unable to access information from 18 nurses who were on leave, but with respect to the large response rate, the missing population should not create a selection bias of any significance. The study covered nurses working in different units in hospitals and primary health care centers. Secondly, this study of Palestinian nurses had a larger proportion (40%) of male nurses in contrast to most studies on nurses from other parts of the world, where females represent a much higher ratio of the nurse population. We used a questionnaire (Pikó, 1999) for assessing the work stress level. The questionnaire was developed in a country (Hungary) that is different from Palestine in several ways; but the subjects belonged to the same profession in both countries, which is a considerable strength.

The question of whether this questionnaire is a valid instrument for assessing PSS may be raised. Responses such as “often back pain” might reflect musculoskeletal disorders rather than psychosomatic problems. However, the association between back pain and self-reported stressful working conditions indicates that this question covers symptoms related to stressful events, whether they are psychosomatic or somatic symptoms.

#### Limitations

Among the limitations are that a cross-sectional design has several potential biases and limitations. Therefore, causality should be interpreted cautiously when drawing conclusions about associations in cross-sectional studies. It is possible that the use of self-report data for exposure (stressful working conditions) and effect (PSS) may have inflated the associations between the exposure and outcome variables (Schnurr & Green, 2004), reducing the reliability of the associations between the exposures and outcomes. Another possible limitation might be that almost all of the nurses in our study were younger than 45 years which may have influenced their reporting of psychological symptoms. A study by Clendon and Walker (2013) reported that nurses aged over 50 consider themselves to have a high health related quality of life.

## Conclusion

The main findings of this study are that women reported more PSS than men, that PSS were associated with perceived self-reported stressful working conditions or work situations and that this association was stronger for men than for women. The study indicates that perceived stressful working conditions can interfere with nurses’ physiological and psychological well-being.

## Practical implications

In Palestine, no psychosomatic questionnaires are available to date. This questionnaire could be a useful instrument for the study of PSS among Arab nurses in the future. Future research using longitudinal designs may validate the findings of this study and provide more information to inform clinical practice and research.

Knowing workplace stressors in clinical areas among nurses help nurse managers and health care administrators to adopt strategies that manage job stressors effectively in work settings such as work scheduling, reduce workload, and improve work environment. Efforts to alleviate stressful working conditions among nurses can lead to an increased quality of care delivery. We recommend to Palestinian nursing policy-makers to choose strategies to help nurses’ cope effectively with workplace stressors. Nursing managers should develop strategies to address and improve the quality of working conditions for nurses. Providing educational and career prospects can contribute to decrease nurses’ occupational stress level, the maintaining their work ability.
